# The NLRP1 and CARD8 inflammasomes

**DOI:** 10.1111/imr.12884

**Published:** 2020-06-19

**Authors:** Cornelius Y. Taabazuing, Andrew R. Griswold, Daniel A. Bachovchin

**Affiliations:** ^1^ Chemical Biology Program Memorial Sloan Kettering Cancer Center New York NY USA; ^2^ Weill Cornell, Rockefeller, Sloan Kettering Tri‐Institutional MD‐PhD Program New York NY USA; ^3^ Tri‐Institutional PhD Program in Chemical Biology Memorial Sloan Kettering Cancer Center New York NY USA; ^4^ Pharmacology Program of the Weill Cornell Graduate School of Medical Sciences Memorial Sloan Kettering Cancer Center New York NY USA

**Keywords:** anthrax lethal toxin, CARD8, DPP8/9, inflammasome, NLRP1, proteasome, pyroptosis, Val‐boroPro

## Abstract

Inflammasomes are multiprotein complexes that activate inflammatory cytokines and induce pyroptosis in response to intracellular danger‐associated signals. NLRP1 and CARD8 are related germline‐encoded pattern recognition receptors that form inflammasomes, but their activation mechanisms and biological purposes have not yet been fully established. Both NLRP1 and CARD8 undergo post‐translational autoproteolysis to generate two non‐covalently associated polypeptide chains. NLRP1 and CARD8 activators induce the proteasome‐mediated destruction of the N‐terminal fragment, liberating the C‐terminal fragment to form an inflammasome. Here, we review the danger‐associated stimuli that have been reported to activate NLRP1 and/or CARD8, including anthrax lethal toxin, *Toxoplasma gondii, Shigella flexneri* and the small molecule DPP8/9 inhibitor Val‐boroPro, focusing on recent mechanistic insights and highlighting unresolved questions. In addition, we discuss the recently identified disease‐associated mutations in NLRP1 and CARD8, the potential role that DPP9’s protein structure plays in inflammasome regulation, and the emerging link between NLRP1 and metabolism. Finally, we summarize all of this latest research and consider the possible biological purposes of these enigmatic inflammasomes.

## 1 INTRODUCTION

**FIGURE 1:**
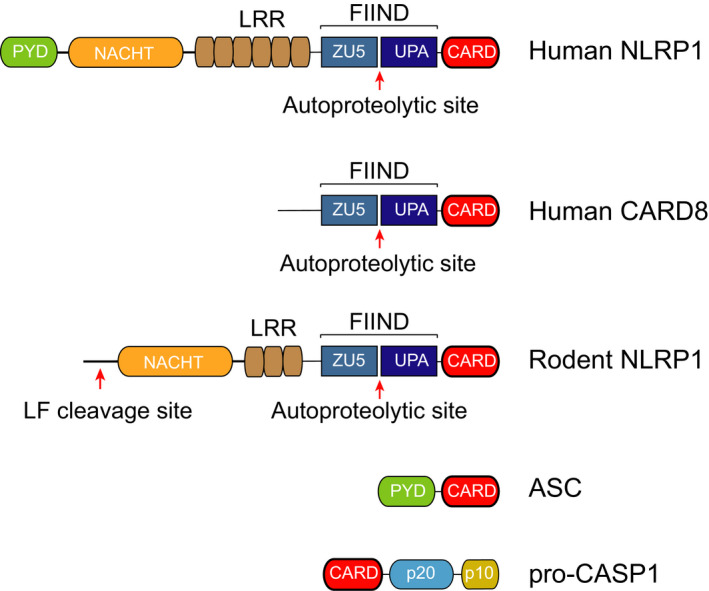
Domain architecture of the NLRP1 inflammasome proteins. NLRP1 and CARD8 protein have FIIND and CARD domains and undergo autoproteolysis between the ZU5 and UPA subdomains that comprise the FIIND. NLRP1 proteins have NACHT and LRR domains preceding the FIIND, and human NLRP1 also has an N‐terminal PYD. Some rodent NLRP1 proteins are cleaved by lethal factor (LF) near their N‐termini. ASC contains a PYD and a CARD, and pro‐CASP1 contains a CARD preceding its catalytic p20 and p10 subunits

Mammals express a number of germline‐encoded pattern recognition receptors (PRRs) that detect and mount immune responses to pathogens.^1^, ^2^ Several of these PRRs are expressed intracellularly and, upon activation, assemble into large multiprotein complexes called inflammasomes.^3^, ^4^ Typically, an inflammasome‐forming PRR recognizes a particular pathogen‐associated structure or activity, oligomerizes and recruits the adaptor protein ASC (apoptosis‐associated speck‐like protein containing a CARD). ASC, which consists of a pyrin domain (PYD) and a caspase activation and recruitment domain (CARD) (Figure 1), bridges either a PYD or a CARD of the activated PRR to the CARD of pro‐caspase‐1 (pro‐CASP1). Next, pro‐CASP1 undergoes proximity‐induced autoproteolysis to generate an active enzyme (CASP1) that cleaves and activates inflammatory cytokines (ie IL‐1β and IL‐18) and gasdermin D (GSDMD).^5^, ^6^, ^7^ The N‐terminal fragment of GSDMD oligomerizes and forms pores in the cellular membrane, triggering an inflammatory form of programmed cell death called pyroptosis. It should be noted that ASC is not always required for inflammasome formation, as some CARD‐containing PRRs can directly recruit pro‐CASP1.^8^, ^9^, ^10^ ASC‐independent inflammasomes still cleave GSDMD and induce pyroptosis, but do not efficiently cleave and activate the inflammatory cytokines.

NLRP1 (nucleotide‐binding domain leucine‐rich repeat pyrin domain containing 1) was the first PRR discovered to form an inflammasome.^11^ In this landmark study, Martinon et al^11^ described the spontaneous assembly of an “inflammasome” complex containing NLRP1 and ASC that activated pro‐CASP1 in immune cell extracts. Since that report, at least five distinct mammalian inflammasomes have been identified, and extensive research has delineated many key aspects of their activation mechanisms.^3^, ^4^ Despite being the first identified inflammasome‐forming PRR, however, NLRP1 remained poorly characterized for many years, and only recent research has started to illuminate its activation mechanism and biological purpose. Here, we review the recent insights into the biology of NLRP1 inflammasome and highlight the key mysteries that remain unsolved.

## 2 NLRP1’s DOMAIN ORGANIZATION

Like the other NLRP proteins, human NLRP1 (hNLRP1) has an N‐terminal pyrin domain (PYD) followed by a NACHT (NAIP, CIITA, HET‐E and TP‐1) domain and leucine‐rich repeats (LRRs) (Figure 1). Unlike the other NLRPs, hNLRP1 has a C‐terminal extension containing a function‐to‐find domain (FIIND) and a CARD. The FIIND consists of ZU5 (found in ZO‐1 and UNC5) and UPA (conserved in UNC5, PIDD and Ankyrin) subdomains and undergoes post‐translational autoproteolysis after its ZU5 subdomain to generate two non‐covalently associated polypeptide chains.^12^, ^13^, ^14^ As described in detail below, FIIND autoproteolysis is required for NLRP1 inflammasome activation.^13^, ^14^ It should be noted that only a fraction (~50%) of the total NLRP1 protein undergoes autoproteolysis,^13^ although it is unknown whether the remaining full‐length protein, which cannot form an inflammasome, has a specific function. The C‐terminal CARD, and not the N‐terminal PYD, recruits ASC to form an inflammasome.^13^ Interestingly, although some CARD domains can directly recruit pro‐CASP1 independent of ASC,^8^, ^9^, ^10^ the hNLRP1 CARD absolutely requires ASC to bridge the interaction with pro‐CASP1.^7^

CARD8 is the only other human protein with a FIIND.^12^ CARD8 and hNLRP1 have similar FIIND‐CARD regions, but CARD8 lacks the structured N‐terminal domains found in NLRP1 (Figure 1). Like hNLRP1, CARD8 undergoes FIIND autoproteolysis^12^ and the C‐terminal CARD domain can form an inflammasome.^15^ However, unlike hNLRP1, the CARD8 CARD directly interacts with the CARD of pro‐CASP1 and does not form an ASC‐containing platform.^7^ The similarities and differences between hNLRP1 and CARD8 are discussed in detail below.

Rodents express homologs of NLRP1 but not CARD8. The mouse genome contains three paralogs of *Nlrp1* (*Nlrp1a,b,c*), although *Nlrp1c* is predicted to be a pseudogene.^16^, ^17^ Unlike hNLRP1, mouse NLRP1A (mNLRP1A) and NLRP1B (mNLRP1B) both lack the N‐terminal pyrin domain (Figure 1) and can recruit pro‐CASP1 either with or without ASC.^10^, ^18^, ^19^ mNLRP1B is extraordinarily polymorphic, with at least five considerably different alleles present in common inbred mouse strains.^17^ mNLRP1B alleles 3 and 4 are non‐functional due to defective autoproteolysis and truncation prior to the CARD, respectively.^14^, ^17^, ^20^ The rat genome contains one *Nlrp1* gene, which, like the mouse *Nlrp1* genes, does not encode an N‐terminal PYD (**Figure** **1**). At least five distinct *Nlrp1* alleles exist in inbred rat strains, although polymorphisms are largely in the first 100 amino acids preceding the NACHT domain and all of the encoded proteins are functional.^20^, ^21^ To our knowledge, the role that ASC plays in the assembly of the rat NLRP1 (rNLRP1) inflammasome has not been established.

## 3 ANTHRAX LETHAL TOXIN

Anthrax lethal toxin (LT) is a bipartite toxin consisting of the pore‐forming protein protective antigen (PA) and the zinc metalloprotease lethal factor (LF). PA transports LF into the host cell cytosol, where it cleaves a number of host proteins, including the mitogen‐activated protein kinase kinases (MAPKKs).^22^, ^23^ In the 1990s, LF protease activity was found to trigger rapid cell death in rodent macrophages.^24^, ^25^, ^26^, ^27^ Notably, proteasome and N‐end rule pathway inhibitors blocked LT‐induced macrophage death,^27^, ^28^, ^29^, ^30^ indicating that the degradation of at least one protein by the N‐end rule machinery was required for cell death to occur.

Interestingly, LT killed macrophages derived from some inbred rodent strains, while macrophages from other rodent strains and humans were completely resistant (Table 1).^24^, ^25^, ^26^, ^27^, ^31^ The susceptibilities of mouse and rat macrophages to LT‐induced death were mapped to the *Nlrp1b* ^17^ and *Nlrp1* ^21^ genes, respectively. Specifically, LT killed macrophages expressing *mNlrp1b* alleles 1 and 5 and *rNlrp1* alleles 1 and 2, suggesting that LT was either directly or indirectly activating only those NLRP1 proteins. It should be noted that macrophage pyroptosis was found to be beneficial to the host, as LT‐sensitive *Nlrp1* alleles provided resistance to *Bacillus anthracis* infection.^32^, ^33^ As the rat NLRP1 alleles mainly differ in their first 100 amino acids, the identity of these residues appeared to be responsible for conferring susceptibility to LT.^21^ Indeed, LF was soon thereafter found to directly cleave the sensitive rNLRP1 allele 2, but not the resistant rNLRP1 allele 5, in this N‐terminal region^34^ (Figure 1). Consistent with direct cleavage stimulating inflammasome assembly, mutation of the LF cleavage site abolished both LF proteolysis and caspase‐1 activation. LF was subsequently found to directly cleave the sensitive mNLRP1B alleles, but not the resistant mNLRP1B alleles, mNLRP1A or hNLRP1.^20^, ^35^, ^36^, ^37^ To determine whether N‐terminal proteolysis could also activate the LF‐resistant NLRP1 variants, Chavarría‐Smith *et al* engineered tobacco etch virus (TEV) protease‐cleavage sites into the N‐terminal regions of mNLRP1B allele 1 and 2, mNLRP1A and hNLRP1 and discovered that TEV protease indeed induced their cleavage and activation.^36^, ^37^ Thus, N‐terminal proteolysis is a general mechanism for NLRP1 activation, even though LF only cleaves a subset of NLRP1 proteins.

**TABLE 1:** Strain/species sensitivity to anthrax LT, *T gondii* and DPP8/9 inhibitors

NLRP1 allele	Strains	Anthrax LT		*T gondii*		DPP8/9 Inhibitor
		MΦ pyroptosis	NLRP1 Cleavage	MΦ pyroptosis	Infection sensitivity	MΦ pyroptosis
*Human*						
N/A	N/A	No	No	High?	Restrictive	Yes
Mouse						
A	N/A	No	No	NT	Permissive^a^	Yes
B1	129S1/SvimJ, BALB/cJ, C3H/HeJ, CBA/J, FVB/NJ, NON/LtJ, NZO/HILtJ, SWR/J	Yes	Yes	Yes	Permissive	Yes
B2	A/J, C57BL/6J, I/LnJ	No	No	Yes	Permissive	Yes
B3	AKR/J, NOD/LtJ, SJL/J (non‐functional)	No	No	NT	NT	No
B4	DBA/2J, P/J, SM/J (non‐functional)	No	NT	NT	Permissive	NT
B5	CAST/EiJ	Yes	Yes	NT	NT	Yes
Rat						
1	BN, WIS, SD, Dahl/SS	Yes	NT	Low	Permissive^b^	Low
2	CDF	Yes	Yes	Low	Permissive	Low
3	ZUC	No	NT	NT	NT	High
4	COP	No	NT	NT	NT	High
5	LEW, WKY, SHR, SHR/Lj	No	No	High	Restrictive^c^	High

Note

In ref. ^50^, OM and DA rats (which express alleles similar to BN) were permissive, and LOU, DBIX and WF (which express alleles similar to LEW) were restrictive. Low and high refer to comparisons between rat strains only. The LT data from refs ^17^, ^20^, ^21^, ^34^, ^35^, ^36^, *T gondii* from refs ^45^, ^48^, ^49^, ^50^, ^51^, ^53^, ^100^, ^101^, ^102^, DPP8/9 inhibitor data from refs ^20^, ^56^, ^103^. NT, not tested.

^a^ Mice strains vary in *T gondii* susceptibility, but are generally more permissive than rats and humans.

^b^ BN, SD tested.

^c^ LEW and SHR tested.

**FIGURE 2:**
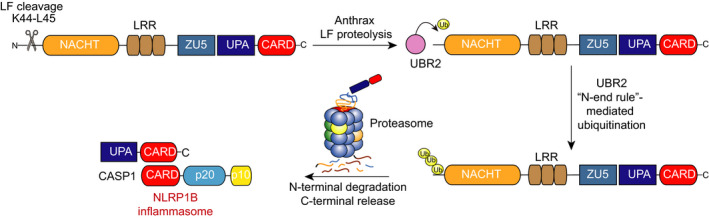
LF activation of the NLRP1B inflammasome. NLRP1B undergoes post‐translational autoproteolysis after its ZU5 subdomain to generate N‐ and C‐terminal fragments that remain non‐covalently associated. LF cleaves between residues K44 and L45 in the N‐terminal fragment, generating an destabilized N‐terminal residue. The N‐end rule E3 ligase UBR2 recognizes and ubiquitinates this neo‐N‐terminus, inducing its proteasome‐mediated degradation. The non‐covalently bound C‐terminal fragment is then freed to recruit and activate pro‐CASP1

The molecular basis of proteolysis‐induced NLRP1 inflammasome activation, however, was still unknown. Specifically, it was not obvious how the removal of a small number of N‐terminal amino acids could result in inflammasome activation nor was it clear why FIIND autoproteolysis, the N‐end rule pathway and proteasome activity were required. The NLRP1 C‐terminal fragment, which contains the inflammasome‐forming CARD, induces pyroptosis when transfected without the N‐terminal fragment.^13^, ^14^, ^38^ Thus, it seemed likely that the N‐terminal fragment was autoinhibitory, and direct proteolysis and proteasome activity somehow relieved this autoinhibition. Last year, two complementary studies discovered that protease cleavage of NLRP1 generates a neo‐N‐terminus that is recognized by the N‐end rule pathway,^39^, ^40^ which ubiquitinates and degrades proteins with destabilizing N‐terminal residues^41^, ^42^ (Figure 2). In particular, the N‐end rule E3 ligase UBR2 recognizes and ubiquitinates the specific neo‐N‐terminus generated by LF cleavage.^39^, ^43^ Importantly, the break in the polypeptide chain within the FIIND domain prevents concomitant degradation of the NLRP1 C‐terminal fragment with the NLRP1 N‐terminal fragment. Instead, the C‐terminal fragment is liberated to recruit and activate pro‐CASP1 and induce pyroptosis. Notably, this model explains why proteasome activity and FIIND autoproteolysis are needed for inflammasome activation. However, it also suggests that some, as yet unknown, mechanisms likely exist to prevent sterile inflammasome activation during normal protein turnover.

**FIGURE 3:**
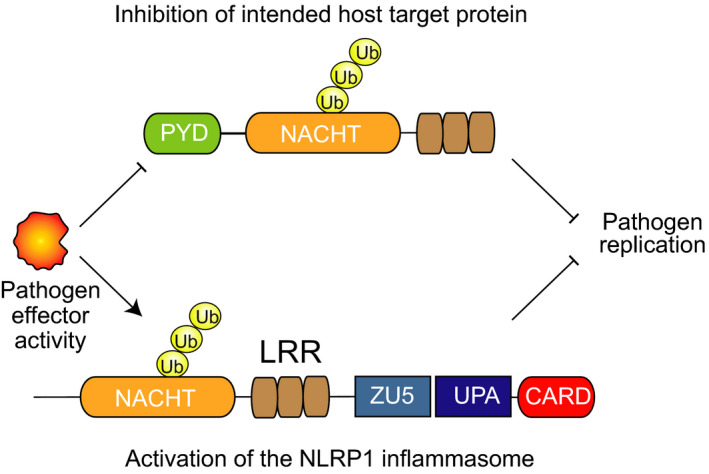
The molecular decoy hypothesis. In principle, pathogen‐derived activities may target host NLR proteins (top) for destruction in order to enhance pathogen replication. NLRP1 (bottom) may act as a decoy for this host protein (or proteins), sensing the destruction of its N‐terminal fragment to induce an immune response

This activation mechanism—dubbed “functional degradation”—suggested that NLRP1 might serve as a “decoy” for other mammalian NLRP proteins^40^, ^44^ (Figure 3). Specifically, pathogens may have evolved a variety of mechanisms, including but not limited to LT, to destroy mammalian NLR proteins and thus evade detection by the innate immune system. However, the accidental destruction of the NLRP1 N‐terminus instead causes inflammasome activation and the induction of an immune response. Consistent with this decoy model, Sandstrom *et al* discovered that the IpaH7.8 E3 ubiquitin ligase secreted by the intracellular bacterial pathogen *Shigella flexneri* directly ubiquitinates and activates mouse NLRP1B allele 1.^40^ As expected, IpaH7.8‐mediated NLRP1B activation required the activity of the E3 ligase and the host proteasome, but, as the IpaH7.8 itself ubiquitinates NLRP1, was independent of UBR2 and the N‐end rule pathway. Future studies are needed to determine whether additional pathogen effectors exist that directly destroy the N‐termini of other NLRP1 proteins, including human NLRP1.

## 4 TOXOPLASMA GONDII

*Toxoplasma gondii* (*T gondii*) is an obligate intracellular parasite that infects a wide range of warm‐blooded animals, although susceptibility to infection varies widely between species and even among individuals of the same species. Generally speaking, rats and humans are far more resistant to *T gondii* infection than mice,^45^, ^46^ but remarkable variation in resistance exists even among inbred rat strains. For example, LEW rats are refractory to infection, but Brown Norway (BN) and Fischer 344 (F344) rats are not.^46^, ^47^ Interestingly, the resistance of LEW rats is a dominant trait, as the progeny of LEW rats and either F344 or BN rats are also resistant to *T gondii*.^46^ Linkage analysis mapped *T gondii* resistance to a 1.7‐cM region termed *Toxo1* that contains the *Nlrp1* gene.^48^

*Toxoplasma gondii* was discovered to induce rapid pyroptosis in LEW and spontaneously hypertensive (SHR) rat bone marrow‐derived macrophages (BMDMs), both of which express *Nlrp1* allele 5 (Table 1).^45^, ^49^, ^50^ In contrast, *T gondii* induced far less pyroptosis in BN and Sprague Dawley (SD) rat BMDMs, which express *Nlrp1* allele 1, and in Fischer (CDF) rat BMDMs, which express *Nlrp1* allele 2 (Table 1). It should be emphasized that *T gondii*‐*resistant* rats have *T gondii*‐*sensitive* macrophages and vice versa, indicating that macrophage pyroptosis is protective against infection. As anticipated by the genetics, *T gondii*‐induced pyroptosis was indeed dependent on NLRP1, as siRNA knockdown of *Nlrp1* in LEW BMDMs reduced cell death and overexpression of the *Nlrp1* allele 5 in CDF macrophages increased cell death.^45^ Overall, these data identified *T gondii* as the second pathogen‐associated activator of the NLRP1 inflammasome after LT. Intriguingly, the rat Nlrp1 alleles that confer susceptibility to *T gondii* are precisely the opposite of those that confer susceptibility to LT, although it is unknown whether this is biologically meaningful or simply coincidence.

As noted above, mice are considerably more susceptible to *T gondii* infection than rats. Consistent with *Nlrp1*‐mediated macrophage pyroptosis playing a critical role in restricting *T gondii* infection, *T gondii* induces far less (and in some assays undetectable) cell death in mouse BMDMs than in rat BMDMs.^45^, ^49^, ^51^ Regardless, Ewald et al^49^ reported that *T gondii* induced at least some pyroptosis in C57BL/6J and 129S1/SvImJ BMDMs and that ectopic expression of the 129 *Nlrp1b* allele 1 in immortalized B57BL/6J BMDMs enhanced *T gondii*‐induced pyroptosis. Moreover, *Casp1/11^−/−^*, *Asc^−/−^* and *Nlrp1^−/−^* mice had reduced survival and higher parasite loads than control mice in response to *T gondii* challenge.^49^, ^51^ Thus, *T gondii* activates at least a few mouse NLRP1 alleles, albeit less strongly than the rat alleles. It should be noted that multiple inflammasomes can signal simultaneously, and *T gondii* infection was also reported to activate the NLRP3 inflammasome in mice.^51^ However, the mechanistic basis of *T gondii*‐induced NLRP3 activation has not been extensively studied.

Preliminary data suggest that the NLRP1 inflammasome may also play a role in restricting *T gondii* infection in humans. Perhaps most notably, polymorphisms in the human *NLRP1* gene were found to be associated with congenital toxoplasmosis.^52^ Surprisingly, however, this same study counterintuitively reported that shRNA‐mediated knockdown of *NLRP1 increased* the amount of *T gondii*‐induced cell death in MonoMac6 cells. A subsequent study found that *T gondii* induced inflammasome activation in human THP‐1 monocytes, although the contribution of NLRP1 to this response was not evaluated.^53^ The role of NLRP1, as well as CARD8, in the response to *T gondii* infection in humans warrants future study.

**FIGURE 4:**
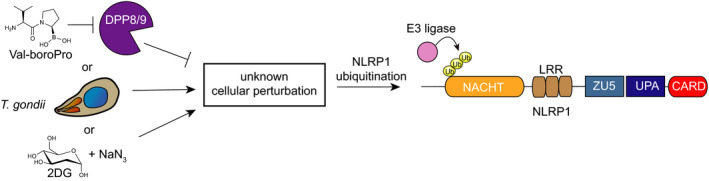
An indirect mechanism to sense pathogen‐associated activities. DPP8/9 inhibitors, *T gondii* infection and metabolic inhibitors may induce the same perturbation within cells, which in turn activates an E3 ligase to ubiquitinate and degrade the NLRP1 N‐terminus. In this model, unlike the “decoy” model, NLRP1 indirectly senses pathogen‐associated activities

The molecular mechanism of *T gondii*‐induced NLRP1 activation is unknown. One possibility is that *T gondii* secretes an effector protein that destroys NLRP1 N‐terminus like LF protease or *S flexneri* IpaH7.8 (Figure 3). Ewald *et al* reported that *T gondii* infection does not cause N‐terminal proteolysis of NLRP1B,^49^ suggesting that, if the decoy model is correct, the key pathogenic effector is not a protease. An alternative possibility is that a *T gondii* activity manipulates the host cell in some way, and NLRP1 senses this perturbation in host cell state (Figure 4). Interestingly, although the NLRP1 alleles vary considerably in their sensitivity to *T gondii*‐induced activation, all alleles tested appear to detect *T gondii* to some degree, unlike LF or IpaH7.8. Thus, it may be likely that *T gondii* triggers a more “universal” activation mechanism than the pathogen effectors that directly act on the NLRP1 protein itself. As described in detailed below, the relative responsiveness of NLRP1 alleles to *T gondii* infection and DPP8/9 inhibition is remarkably similar, suggesting a possible shared activation mechanism.

## 5 THE DIPEPTIDYL PEPTIDASES 8 AND 9 (DPP8/9)

The small molecule Val‐boroPro (VbP, Figure 4) was discovered to stimulate anti‐cancer immune responses in syngeneic mouse models more than 15 years ago.^54^, ^55^ However, the molecular mechanism of immune activation remained enigmatic until recently. In 2017, VbP was found to induce caspase‐1‐dependent pyroptosis in a number of human and mouse cell types, including human THP‐1 monocytes, human peripheral blood mononuclear cells (PBMCs), mouse RAW 264.7 macrophages and primary mouse BMDMs.^56^, ^57^ Importantly, caspase‐1 was required for VbP’s immunostimulatory activity in animals, demonstrating that pyroptosis was critical for its intriguing anti‐cancer effects.

VbP is a non‐selective inhibitor of the post‐proline cleaving serine proteases, with particularly potent activity against the dipeptidyl peptidases DPP4, DPP7, DPP8 and DPP9.^58^ *DPP9*, but not *DPP4, DPP7* or *DPP8*, knockout in THP‐1 cells caused cell death, suggesting that DPP9 was the key VbP target that restrained pyroptosis. However, VbP still induced some cell death in *DPP9^−/−^* THP‐1 cells, indicating at least one additional VbP‐sensitive enzyme supported cell viability in the absence of DPP9. DPP8 was hypothesized to be this target, as it is the most similar protein to DPP9 and, like DPP9, is localized in the cytosol.^59^ Indeed, *DPP8/9^−/−^* THP‐1 cells were completely resistant to VbP, and selective (dual) DPP8/9 inhibitors also induced pyroptosis. It should be noted that, due to the structural similarity of their active sites, no inhibitors selective for DPP9 over DPP8 or vice versa have been identified.

The identity of the VbP‐activated inflammasome remained unknown. However, ASC was reported to be dispensable for VbP‐induced pyroptosis in human THP‐1 cells and mouse RAW 264.7 macrophages,^56^ suggesting that DPP8/9 inhibition activates a CARD‐containing PRR that can directly recruit pro‐CASP1 in those cell types.^56^ As mentioned above, mNLRP1A and mNLRP1B do not require ASC to bridge to pro‐CASP1,^10^, ^18^, ^19^ making these likely candidates.^60^ RAW 264.7 cells were derived from BALB/c mice, which express NLRP1B allele 1 and do not express NLRP1A.^16^, ^61^ Okondo et al^60^ thus created *Nlrp1b^−/−^* cells and found that they, like *Casp1^−/−^*, RAW 264.7 cells were completely resistant to VbP and selective DPP8/9 inhibitors. Similarly, *Nlrp1‐*deficient (lacking both NLRP1A and B) primary mouse BMDMs were resistant to VbP, and VbP failed to induce cytokines in *Nlrp1‐*deficient mice. Since this report, DPP8/9 inhibitors have been shown to activate NLRP1A, the three functional NLRP1B alleles and all five rat NLRP1 alleles, thus becoming the first known activator of all of the rodent NLRP1 inflammasomes.^20^ Intriguingly, the various rat NLRP1 alleles have remarkably different sensitivities to VbP, perfectly mirroring their relative sensitivities to *T gondii* (Table 1). Thus, it seems possible that *T gondii* and VbP may share the same activation mechanism, as discussed further below.

Interestingly, Johnson *et al* discovered that VbP induced caspase‐1‐dependent pyroptosis in a number of acute myeloid leukaemia (AML) cancer cell lines, including MV4;11 and OCI‐AML2 cells, in addition to THP‐1 cells.^15^ Surprisingly, CARD8, and not hNLRP1, was found to mediate VbP‐induced pyroptosis in these cells,^15^ identifying CARD8 for the first time as an inflammasome‐forming PRR. Although VbP did not activate NLRP1 in these cancer cell lines, perhaps due to low NLRP1 expression, VbP was subsequently found to induce NLRP1‐dependent pyroptosis in keratinocytes.^62^ As such, VbP not only activates all functional rodent alleles, but also hNLRP1 and CARD8, and thus appears to be a universal NLRP1 activator.^20^

The mechanistic basis of NLRP1 and CARD8 activation by DPP8/9 inhibition is an area of active research. The ectopic expression of CARD8 or mNLRP1 together with CASP1 and GSDMD renders HEK 293T sensitive to VbP, indicating that all of the other key proteins needed to execute this pyroptotic pathway are endogenously present in HEK 293T cells. hNLRP1 also requires the co‐expression of ASC in order to bridge to CASP1.^7^, ^13^ Like LF, VbP induces the proteasome‐dependent N‐terminal degradation of the sensitive PRRs, releasing their C‐terminal fragments to form inflammasomes.^15^, ^39^ Unlike LF but like *T gondii*, VbP does not appear to cause the direct cleavage of the N‐terminal fragments.^15^, ^60^ Consistent with this observation, VbP‐induced pyroptosis is not dependent on the N‐end rule pathway.^39^, ^60^

DPP8/9 cleave N‐terminal dipeptides, and in particular those with proline in the second position (NH_2_‐Xaa‐Pro), from polypeptide substrates.^63^, ^64^ Interestingly, the neo‐N‐terminus of the NLRP1 C‐terminal fragment is NH_2_‐Ser‐Pro, raising the possibility that DPP8/9 directly cleaves NLRP1 itself to restrain inflammasome assembly. However, a study using Chemical Enrichment of Protease Substrates (CHOPS) found that the mNLRP1B allele 1 C‐terminal fragment is not a direct DPP8/9 substrate,^65^ and immunoprecipitation‐mass spectrometry (IP‐MS) experiments failed to identify an N‐terminal peptide consistent with DPP8/9 cleavage of hNLRP1.^62^ Moreover, the N‐terminal sequence of CARD8’s neo‐C‐terminal fragment is NH_2_‐Ser‐Leu, which is not a preferred DPP8/9 substrate. Taken together, these data indicate that DPP8/9 do not restrain the NLRP1 and CARD8 inflammasomes by direct cleavage.

It is possible that some, as yet unknown, DPP8/9 substrate(s) regulate NLRP1 and CARD8 activation.^56^, ^62^ Intriguingly, DPP9 substrate profiling studies using terminal amine isotopic labelling of substrates (TAILS) in SKOV3 cells^66^ and CHOPS in THP‐1 cells^65^ identified very few potential protein substrates. Instead, CHOPS analysis indicated that DPP8/9 preferentially cleaved after proline residues in unstructured peptides, rather than globular proteins. Consistent with this result, DPP9 has been reported to catalyse the rate‐limiting step in the catabolism of proline‐containing peptides generated by the proteasome.^67^ Based on these data, it is tempting to speculate that inhibition of DPP8/9‐mediated peptide cleavage induces a cellular perturbation (perhaps the same one that *T gondii* induces) that is indirectly sensed by NLRP1 and CARD8 (Figure 4).^20^ Future studies are needed to explore this hypothesis and clarify the function of DPP8/9’s catalytic activity.

**FIGURE 5:**
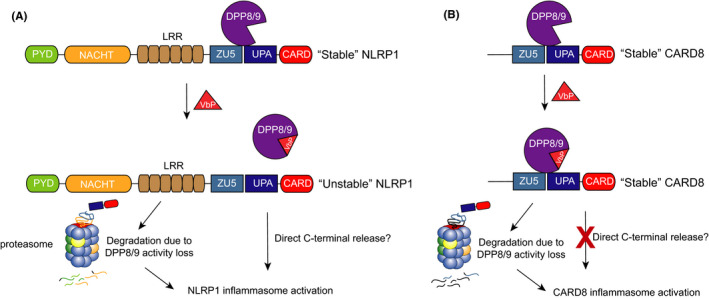
Contribution of DPP9 binding to hNLRP1 and CARD8 activation. Inhibition of DPP8/9’s catalytic activity induces the degradation of the hNLRP1 and CARD8 N‐termini, triggering inflammasome assembly. In addition, DPP9 binds to the FIINDs of both hNLRP1 and CARD8, potentially in order to help stabilize the proteins. VbP disrupts the hNLRP1‐DPP9 (A) but not CARD8‐DPP9 (B), interaction. Direct displacement may contribute to hNLRP1 inflammasome activation by destabilizing hNLRP1 and releasing its C‐terminus from autoinhibition

In addition to its catalytic activity, DPP9’s protein structure also appears to play a role in restraining inflammasome activation. Notably, DPP9 (as well as DPP8) was recently discovered to directly bind the FIINDs of both hNLRP1 and CARD8.^62^, ^68^ The catalytic activity of DPP9 was not required for these interactions, as the catalytically dead S759A mutant DPP9 still bound to both hNLRP1 and CARD8. Despite these similarities, however, the hNLRP1‐DPP9 and CARD8‐DPP9 interactions appear to be remarkably distinct (Table 2). First, ectopically expressed wild‐type and autoproteolysis‐defective mutant CARD8 bound equally well to DPP9 in HEK 293T cells.^62^, ^68^ In stark contrast, autoproteolysis‐defective mutant hNLRP1 exhibited significantly impaired binding in this assay. Thus, autoproteolysis is required only for the hNLRP1‐DPP9 interaction. Second, DPP8/9 inhibitors partly reduced the binding of DPP9 to hNLRP1, but not to CARD8^62^, ^68^ (Figure 5). These data strongly suggest that the hNLRP1 binding interface involves at least some surface that is proximal to the DPP9 active site, whereas the CARD8 binding interface appears to be entirely spatially distant from the DPP9 active site. Consistent with this premise, an extended activity‐based probe (MW = 1087) was found to react with the catalytic serine of DPP9 without displacing the CARD8‐DPP9 interaction.^68^ Interestingly, it should be noted that mNLRP1B was also reported to be a direct binding partner of DPP9.^68^ Surprisingly, however, the mNLRP1B‐DPP9 interaction more closely resembled the CARD8‐DPP9 interaction than the hNLRP1‐DPP9 interaction, as it did not require FIIND autoproteolysis and was not disrupted by VbP.^68^

**TABLE 2:** Summary of hNLRP1, CARD8 and mNLRP1B binding to DPP9

Binding Interaction	hNLRP1‐DPP9	CARD8‐DPP9	mNLRP1B‐DPP9
Subdomains required	ZU5‐UPA	ZU5‐UPA	NT
Binding to catalytically inactive DPP9	Yes	Yes	NT
Binding of autoproteolysis deficient mutant^a^	Minimal	Yes	Yes
VbP displacement in vitro	Yes	No	No
Catalytically inactive DPP9 inhibits the inflammasome	Yes^b^	No^c^	NT

^a^ Autoproteolysis deficient mutants tested include: hNLRP1 F1212A and S1213A, CARD8 S297A, and mNLRP1B S984A.

^b^ Stable expression of catalytically inactive DPP9 S759A partially rescued NLRP1‐ASC puncta formation in reconstituted DPP8/9^−/−^ HEK 293T cells.^62^, ^68^

^c^ Stable expression of DPP9 S759A did not rescue CARD8‐CASP1 mediated cell death in reconstituted DPP9^−/−^ HEK 293T cells.^66^

Although the functional importance of these binding interactions has not yet been full established, preliminary data suggest that DPP9 binding restrains hNLRP1 inflammasome formation. For example, the expression hNLRP1 and ASC in *DPP8/9^−/−^*, but not wild‐type, HEK 293T cells caused the formation of spontaneous ASC specks. In this system, stable expression of wild‐type DPP9, which both binds to hNLRP1 and has catalytic activity, completely abrogated speck formation. Notably, the stable expression of DPP9 S759A, which binds to hNLRP1 but is catalytically inactive, partially rescued speck formation.^62^ Consistent with these data, a hNLRP1 P1214R mutant protein (discussed below) that is unable to bind to DPP9 spontaneously assembles into an inflammasome.^62^, ^69^ Additional research is needed to elucidate how DPP9 binding prevents inflammasome activation at a molecular level, but it seems plausible that DPP9 may either stabilize the association of the two NLRP1 fragments or help regulate non‐inflammatory, basal NLRP1 protein turnover. Regardless, based on these data, it appears that DPP8/9 inhibitors likely activate hNLRP1 by *both* inhibiting DPP8/9 activity and disrupting the DPP9‐hNLRP1 interaction (Figure 5).

In a separate study, Griswold *et al* reported that *DPP9^−/−^* HEK293T cells spontaneously died when expressing CASP1 and CARD8, and that VbP did not further increase the amount of cell death. In this system, stable expression of wild‐type DPP9 rescued cell death and re‐sensitized the cells to VbP, as expected. However, stable expression of DPP9 S759A, which retains CARD8 binding but is catalytically inactive, did not rescue cell death. These data indicate that DPP9 binding does not significantly restrain inflammasome activation *in this system*. We should emphasize that this does not definitively rule out a role for DPP9 binding in restraining CARD8 (Figure 5), but rather suggests that binding cannot fully compensate for the loss of DPP9’s catalytic activity in HEK 293T cells. Regardless, since VbP does not disrupt the DPP9‐CARD8 interaction, DPP8/9 inhibitors likely activate the CARD8 inflammasome exclusively by blocking enzymatic activity. Future studies are needed to determine how and why hNLRP1 and CARD8 interact differently with DPP9.

## 6 METABOLIC INHIBITORS

Mogridge and co‐workers, noting the link between infectious disease and host metabolism, investigated the relationship between energy stress and NLRP1B activation.^61^, ^70^, ^71^ Liao et al^70^ initially evaluated the impact of glycolysis and oxidative phosphorylation inhibitors on reconstituted inflammasome systems in HT1080 human fibroblasts. Briefly, HT1080 cells were transfected with constructs encoding an NLR protein, pro‐caspase‐1 and pro‐IL‐1β before being treated with the glycolysis inhibitor 2‐deoxyglucose (2DG) and the electron transport chain inhibitor sodium azide. Intriguingly, IL‐1β release was observed in cells expressing the functional NLRP1B allele 1, but not the autoproteolysis‐defective NLRP1B allele 3, NLRP3 or NLRP6, indicating that this inhibitor combination specifically activated NLRP1B. Consistent with a “functional degradation” mechanism, proteasome inhibition also blocked IL‐1β release. Notably, like *T gondii* and DPP8/9 inhibitors, 2DG plus sodium azide activated NLRP1B without direct N‐terminal cleavage.^70^, ^71^

Although the molecular details leading to NLRP1B activation remain largely unknown, the authors speculated that 2DG plus sodium azide activates NLRP1B via depletion of cytosolic ATP. Supporting this hypothesis, cellular ATP levels were inversely correlated with inflammasome activation, and other perturbations that lower ATP, including hypoxia and glucose‐free media, also activated NLRP1B in the HT1080 system. As NACHT domains bind ATP,^72^ the authors postulated that NLRP1B might directly sense ATP levels. Intriguingly, mutations in the Walker A site of the NACHT domain, which plays a critical role in ATP binding, generated a constitutively active NLRP1B protein.^70^ Thus, it is possible that a loss of ATP in the NACHT domain results in inflammasome formation. However, it is also possible that the Walker A mutation simply destabilizes the N‐terminus of the protein, leading to its increased degradation by the proteasome.

Recently, the Mogridge lab reported that high doses of 2DG, *Listeria monocytogenes* and *S flexneri* activate the NLRP1B inflammasome in RAW 264.7 cells.^61^ Like 2DG, *L monocytogenes* and *S flexneri* reduced cytosolic ATP levels, and the authors thus speculated that these stimuli may all activate NLRP1B via ATP depletion. However, it is possible that other bacteria‐associated processes, including, for example, direct ubiquitination of NLRP1B by *S flexneri* IpaH7.8,^40^ are triggering this inflammasome assembly. Nevertheless, these findings suggest that NLRP1B may sense a metabolic disturbance, and perhaps this same disturbance is induced by DPP8/9 inhibition and *T gondii* (Figure 4).

## 7 NLRP1 AND CARD8 IN HEALTH AND DISEASE

The first hyperactivating NLRP1 mutation was discovered in mice.^19^ In this study, Masters et al performed an *N*‐ethyl‐*N*‐nitrosourea (ENU) mutagenesis screen and identified a pedigree, dubbed *Neut1*, with a glutamine‐to‐proline point mutation (Q593P) in the *Nlrp1a* gene that caused multiorgan neutrophilic inflammatory disease. Notably, NLRP1A^Q593P^‐induced disease required CASP1 and the interleukin‐1 receptor (IL‐1R), and LPS priming of BMDMs from *Nlrp1a^Q593P/Q593P^* mice, but not control mice, elicited the release of processed IL‐1β. Thus, this mutation appeared to generate a constitutively active, or at least more easily activated, form of NLRP1A. It has not yet been established how the Q593P mutation dysregulates NLRP1A, but it is possible that this mutation, which is located between the NACHT and LRR domains, weakens the autoinhibitory activity of the N‐terminal fragment or increases its propensity to be degraded by the proteasome.

Interestingly, IL‐18 deficiency leads to obesity, insulin resistance and metabolic syndrome in mice.^73^, ^74^ In 2016, Murphy et al^75^ reported that the NLRP1 inflammasome was responsible for generating this IL‐18. Briefly, *Nlrp1^−/−^* mice, like *IL18^−/−^* mice, developed spontaneous obesity and metabolic syndrome. In contrast, *Nlrp1^Q593P/Q593P^IL1r^−/−^* mice (*IL*‐*1r* knockout was needed to prevent inflammatory pathology) weighed less and had reduced adipose tissue mass compared to control animals. Moreover, these animals, when placed on a high‐fat diet (HFD), did not gain weight and died of cachexia before 15 weeks of age. These mutant mice had higher levels of plasma IL‐18, and deletion of *IL18* in these animals abolished weight loss and cachexia. Thus, NLRP1 appears to play a role in preventing obesity and metabolic syndrome in mice via the production of IL‐18. Overall, these observations further highlight the emerging link between the NLRP1 inflammasome and metabolism.

In humans, polymorphisms in the *NLRP1* gene and nearby regions have been associated with a number of autoimmune and autoinflammatory diseases,^76^ including vitiligo,^77^, ^78^ Addison's disease^79^, ^80^ and caeliac disease.^81^ For example, L155H and M1184V are two polymorphisms that are inherited together due to linkage disequilibrium and are associated with high risk for vitiligo and autoimmune disease. Consistent with this risk, PBMCs from subjects with this haplotype released greater amounts of processed IL‐1β relative to the reference haplotype.^82^ Interestingly, NLRP1 M1184V underwent FIIND autoprocessing to a greater extent than wild‐type NLRP1 when overexpressed in HEK 293T cells, suggesting a potential molecular mechanism for increased activity.^13^ However, it should be noted that this polymorphism is not sufficient to cause disease on its own, and thus, additional factors also contribute to the development of autoimmune disease in these individuals.

**FIGURE 6:**
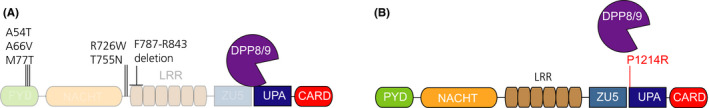
Mutations in hNLRP1 that cause autoinflammatory disease. A, The indicated mutations in the N‐terminal fragment of hNLRP1 potentially destabilize this fragment or interfere with its ability to inhibit the C‐terminal fragment. Transparency is used to indicate the possible increased proteasome‐mediated degradation of this fragment. B, The P1214R mutation, which is located immediately after the autoproteolysis site, disrupts the DPP9 binding interaction and causes spontaneous inflammasome activation

A number of rare gain‐of‐function mutations in the human *NLRP1* gene were discovered to cause skin inflammatory and cancer susceptibility syndromes in 2016.^38^ In this study, Zhong *et al* used whole‐exome sequencing to identify the causal mutations in multiple self‐healing palmoplantar carcinoma (MSPC) and familial keratosis lichenoides chronica (FKLC), two diseases that present with similar skin‐related inflammatory pathologies. Interestingly, individuals with MSPC had missense mutations in the PYD of NLRP1 (A54T, A66V M77T), and individuals with FKLC had an in‐frame deletion (F787‐R843) that removed the first LRR domain and part of the preceding linker region (Figure 6A). As expected, these mutations were confirmed to increase inflammasome activation. Notably, the MSPC mutations appeared to disrupt the PYD folding, as determined by 2D[15^N,1^H]‐HSQC NMR and circular dichroism analyses. Thus, it seems likely that these PYD mutations destabilize the N‐terminal fragment, increasing its susceptibility to degradation by protein quality control pathways.^44^ The molecular basis of inflammasome activation by the FKLC deletion has not been extensively studied, but it might similarly destabilize the N‐terminal fragment or in some other way weaken its autoinhibitory activity.

A subsequent analysis of three patients from two unrelated families presenting with autoinflammation with arthritis and dyskeratosis (AIADK) identified two additional mutations in NLRP1 (R726W and P1214R).^69^ In addition, a homozygous gain‐of‐function mutation in NLRP1 (T755N) was found in siblings with a syndromic form of juvenile‐onset recurrent respiratory papillomatosis (JRRP).^83^ As mentioned above, the P1214R mutation was found to abrogate binding to DPP9.^62^ Importantly, these data strongly suggesting that DPP9 binding indeed serves to stabilize the autoinhibited form of NLRP1 and that disruption of this binding interaction leads to inflammasome activation (Figure 6B). The mechanistic basis of the NLRP1 R726W and T755N mutations have not been extensively studied, but N‐terminal destabilization or weakened autoinhibitory activity are likely to be involved.

The role that CARD8 plays in human health and disease is poorly understood. Several studies linked a polymorphism that creates a stop codon at residue 10 (p.C10X) in the T48 isoform of CARD8 to inflammatory bowel disease^84^, ^85^, ^86^ and rheumatoid arthritis,^87^, ^88^ but other studies have questioned this finding.^85^, ^89^, ^90^, ^91^ It is worth noting that numerous isoforms of CARD8 have been reported with different N‐terminal regions and that CARD8 expression has been observed in homozygotes for this stop allele.^92^ As such, this stop codon may not, in fact, prevent protein expression. Recently, a frameshift variant in *CARD8* that creates a premature stop codon in all CARD8 isoforms was reported to be associated with periodic fever with aphthous stomatitis, pharyngitis and cervical adenitis (PFAPA) syndrome.^93^ This truncated protein lacks the FIIND‐CARD region and is likely non‐functional, but additional studies are needed to evaluate the relevance of this frameshift mutation to PFAPA. Lastly, a mutation (V44I) in the longest CARD8 isoform (T60) was recently identified in three individuals with Crohn's disease.^94^ The V44I mutation appeared to interfere with CARD8’s reported ability to downregulate NLRP3 activation,^95^ but more studies are needed to determine whether NLRP3 regulation is really a critical function of CARD8. Interestingly, CARD8 V44I appeared to oligomerize with itself more robustly than wild‐type CARD8 in HEK 293T cells, potentially indicating a mechanism of hyperactivation. Overall, more research is needed to confirm the functional relevance of these CARD8 mutations in autoimmune disease.

Collectively, the research over the past five years has strongly suggested that NLRP1 and CARD8 are potential targets for therapeutic development. On the one hand, inhibitors of these inflammasomes would likely counteract a number of autoinflammatory diseases, particularly those with skin‐related pathologies. Unfortunately, no direct or indirect inhibitors of these inflammasomes have been reported yet, but such compounds would certainly be of great interest. On the other hand, the pharmacological activation of these inflammasomes holds promising anti‐cancer potential. As mentioned above, VbP itself induces anti‐cancer responses in syngeneic mouse models.^54^, ^55^ It is possible that a more selective DPP8/9 inhibitor, or specific combinations of DPP8/9 inhibitors with other agents, will further increase the efficacy of this immuno‐oncology strategy. In addition, DPP8/9 inhibitors directly kill cancer cell expressing the key inflammasome components. For example, VbP induces CARD8‐mediated pyroptosis in AML cells in vivo, slowing cancer progression.^15^ Thus, the anti‐cancer potential of NLRP1 and CARD8 inflammasome activation also warrants further study.

## 8 CONCLUSIONS AND FUTURE DIRECTIONS

Although NLRP1 was discovered to form an inflammasome in 2002, it remained poorly characterized for many years in large part due to a lack of bona fide activators. Fortunately, a number of NLRP1 activators, including the LF protease, *S flexneri* IpaH7.8, *T gondii* infection and VbP, have now been discovered. Interestingly, these stimuli can be divided into two groups. LF and IpaH7.8 belong to one group—the “direct activators”—that directly modify and degrade the NLRP1 N‐terminal fragment. Notably, these agents only activate a subset of NLRP1 alleles. VbP and potentially *T gondii* and metabolic inhibitors belong to the other group—the “indirect activators”—that appear to induce some cellular disturbance that apparently all NLRP1 and CARD8 proteins detect. Interestingly, these two groups suggest starkly different biological purposes of the NLRP1 inflammasome.

As described above, the direct activators raise the possibility that NLRP1 exists as a “molecular decoy” for other innate immune receptors (Figure 3). This model proposes that a number of distinct pathogen effectors have evolved to degrade host‐derived proteins, including the NLR protein family, that normally inhibit pathogen replication. However, these effectors also accidently destroy NLRP1’s N‐terminus, which closely resembles the intended targets, and trigger immune responses. This model also offers a possible explanation for highly polymorphic nature of NLRP1, as a decoy protein that detects a wide variety of constantly evolving effectors would be under intense selection pressure. It should be noted that decoy receptors have been observed in plants,^96^, ^97^ and thus, this mechanism is not entirely unprecedented. However, more research is needed to confirm that NLRP1 really acts as a decoy. Most importantly, there is no evidence yet that the pathogen effectors that directly degrade NLRP1 also degrade other NLR proteins (ie the intended targets), as this model predicts. In fact, LF itself cleaves NLRP1 in an unstructured region that is not present in other NLRs, arguing that LF was not evolved to destroy NLRs. Of course, it is possible that the intended targets include proteins in addition to NLRs and that the relationships between these targets and the NLRP1 decoy have not yet been discovered.

Alternatively, the indirect activators suggest that NLRP1’s principal function might be to monitor cellular homeostasis (Figure 4). Specifically, VbP and *T gondii* potentially disturb homeostasis in the same way, which in turn activates an unknown host E3 ligase to degrade the NLRP1 N‐terminus.^20^ Given the established relationships between NLRP1,^75^ DPP enzymes^98^ and *T gondii* ^99^ with metabolism, a provocative possibility is that interference with some specific aspect of cell metabolism initiates inflammasome assembly. Notably, the reports that glycolysis and oxidative phosphorylation inhibitors activate mNLRP1B in at least some contexts further supports this idea.^61^, ^70^, ^71^ More research is needed to identify this potential perturbation and the responsive E3 ligase. On that note, it will also be important to determine the molecular features of the very different NLRP1 and CARD8 N‐termini that mediate E3 ligase recognition. It is tempting to speculate that the general recognition features are similar for both proteins, but that the domains of NLRP1 modulate its accessibility. As NLRP1 forms an ASC‐containing inflammasome that likely generates a more intense immune reponse,^7^ it is not unlikely that more elements regulate NLRP1 activation than CARD8 activation.

In summary, a number of recent studies have significantly advanced our understanding of the NLRP1 and CARD8 inflammasomes. However, several unresolved mysteries remain, including the most important one of all: the biological purpose of these proteins. Intriguingly, some lines of evidence have suggested that NLRP1 may act as a molecular decoy to guard other innate immune receptors, while others have indicated that NLRP1 might monitor the cell's metabolic state. It will be of great interest to further explore these possibilities and ultimately define the role that these inflammasomes play in host defence.

## CONFLICT OF INTEREST

The authors declare that they have no conflict of interest.

## AUTHOR CONTRIBUTIONS

DAB, CYT, and ARG wrote this review.
